# Single-cell transcriptome revealed the aberrant keratinocytes activation in antigen presentation in atopic dermatitis

**DOI:** 10.1080/07853890.2026.2627742

**Published:** 2026-02-10

**Authors:** Wen-Xiang Liu, Yan Cao, Yi-Fei Chen, Yi-Fan Lu, Chun-Yu Xu, Yan-Hong Zhai, Cheng Wang, Zheng Cao

**Affiliations:** a Department of Laboratory Medicine, Beijing Obstetrics and Gynecology Hospital, Capital Medical University, Beijing Maternal and Child Health Care Hospital, Beijing, China; b Institute of Traditional Chinese Medicine Pharmacology, Shandong Academy of Chinese Medicine, Jinan, China; c Center of Clinical Mass Spectrometry, Beijing Obstetrics and Gynecology Hospital, Capital Medical University, Beijing Maternal and Child Health Care Hospital, Beijing, China; d Department of Gynecology Oncology, Beijing Obstetrics and Gynecology Hospital, Capital Medical University, Beijing Maternal and Child Health Care Hospital, Beijing, China

**Keywords:** Atopic dermatitis, single-cell RNA sequencing, keratinocytes, antigen presentation, inflammation

## Abstract

**Background:**

Atopic dermatitis (AD), a common chronic inflammatory skin disease, has been extensively studied using single-cell genomics. However, keratinocytes, as key effector cells in AD, have underlying mechanisms remain incompletely understood and require further investigation.

**Methods:**

We integrated single-cell transcriptomic data from skin tissues of healthy controls, chronic active AD patients, spontaneously healed AD (SHAD) patients, and an ovalbumin-induced AD mouse model. The study particularly emphasized the gene expression and cellular dynamics of keratinocytes across the different groups, as well as their interactions with immune cells.

**Results:**

Compared to healthy controls, we observed significant changes in the keratinocyte transcriptome, cellular state, and keratinocyte-immune cell ligand-receptor interactions in AD skin, particularly the marked activation of genes involved in antigen processing and presentation. Interestingly, such gene activation was not observed in keratinocytes from the ovalbumin-induced AD mouse model, despite its phenotype closely resembling human AD. Furthermore, in SHAD, we identified a recovery of both the ligand-receptor interaction patterns and antigen processing and presentation genes, accompanied by a notable shift in the transcriptome. This involved a significant downregulation of genes related to cytoplasmic transcription and oxidative phosphorylation. Notably, this pattern was not observed in the self-healing mouse model following the removal of ovalbumin stimulation.

**Conclusion:**

Our results suggest that the persistent activation of antigen processing and presentation pathways in keratinocytes may be a key driver of chronic inflammation in AD. Therefore, redirecting anti-allergic therapeutic strategies from solely targeting immune cells to targeting of keratinocyte-mediated antigen presentation may offer a more effective approach. Furthermore, we raise concerns about the use of ovalbumin-induced mouse models to recapitulate human chronic AD, as the underlying mechanisms may differ significantly.

## Introduction

Atopic dermatitis (AD) is a common complex chronic, inflammatory and relapsing skin disease, with a prevalence of approximately 20%, particularly in children but also in adults [1,2]. As patients age, approximately two-thirds experience spontaneous remission during adolescence [3], while the remaining individuals continue to suffer from a chronic relapsing course in later years, which is often refractory to treatment [4]. The mechanisms underlying remission in AD and the precise pathological causes of persistent disease remain unclear. The pathophysiology of AD is complex, involving genetic predisposition, skin barrier dysfunction, and abnormal immune activation [1,5]. Immune dysregulation is a central feature of AD [6], and most therapeutic agents target immune cells, cytokines, and their associated signalling pathways, with a particular focus on Th2-driven pathways [7,8]. Abnormal keratinocytes are typically viewed as a passive consequence of the immune response in patients with AD, which has limited research into their active role in driving chronic inflammation.

The use of animal models has advanced our understanding of the chronic nature and long-term progression of AD [9]. However, given the complexity and duration of the disease [10], it remains uncertain whether these models fully recapitulate the pathophysiological features of AD. Additionally, the chronic and relapsing nature of AD poses considerable challenges for the development of targeted therapies in clinical practice [11].

In the immune response of AD, keratinocytes play a crucial role as the first line of defence [12]. Keratinocytes are not only involved in maintaining the skin barrier but also regulate immune cell migration, polarization, and plasticity by secreting cytokines and chemokines, thereby modulating the skin’s immune response [13–15]. Despite their critical involvement, research into the specific contribution of keratinocytes to chronic inflammation in AD has been limited, and no targeted therapies addressing keratinocytes have been developed [11]. Therefore, this study focuses on characterizing transcriptional and functional changes in keratinocytes in AD, with an emphasis on their roles in antigen processing and presentation and on evaluating how well current mouse models capture these mechanisms.

Recent advances in single-cell RNA-seq (scRNA-seq) have provided deeper insights into the pathogenesis of AD and the molecular and cellular mechanisms underlying its progression [16]. By integrating scRNA-seq data from AD and spontaneously healed samples, we can not only deepen our understanding of potential therapeutic mechanisms but also explore the underlying causes of its chronic relapsing nature [8]. Furthermore, scRNA-seq enables a detailed comparison of cellular and molecular features between mouse models and human diseases, highlighting key differences in immune responses, cell composition, and gene expression [17]. This helps identify the most suitable animal models for research and accelerates the development of targeted therapies.

In our study, we integrated scRNA-seq data from human AD, spontaneously healed AD (SHAD), and ovalbumin (OVA)-induced AD mouse models to explore the role of keratinocytes in chronic inflammation. Our findings suggest that keratinocyte-mediated antigen processing and presentation are key drivers of the chronic-recurrent inflammatory nature of AD. Notably, significant differences between the OVA-induced mouse model and human AD, particularly in the keratinocyte transcriptome and chronic self-healing dynamics, limit the use of this model for validating keratinocyte-targeted therapies. In conclusion, this study underscores the critical role of keratinocytes in AD inflammation and the limitations of OVA-induced models in studying its pathogenesis, especially considering the abnormal activation of keratinocytes in the disease.

## Materials and methods

### Animals

All animal experiments in this study were approved by the Animal Ethics Committee of Shandong Academy of Chinese Medicine and conducted following standard protocols (SDZYY20240304004). The BALB/c mice aged 6 weeks (18–22 g) were purchased from Jinan Pengyue Laboratory Animal Breeding Co., Ltd. and housed under specific pathogen-free conditions with controlled temperature and humidity, free access to food and water, and a 12-hour light/dark cycle.

After induction of anaesthesia with 2-2.5% isoflurane (RWD Life Science Co., Ltd., R510-22-10) in an oxygen mixture, six-week-old mice underwent dorsal hair depilation. A total of 15 mice were then randomly assigned to three groups (*n* = 5 per group) [18,19]: the control group (CON), the OVA-induced model group (OVA), and the spontaneous healing model group (SHOVA). Each individual animal was considered as the experimental unit. Mice in the CON group received saline treatment every other day for 12 days before sampling as described previously [18]. Mice in the OVA group were treated with 200 μg of ovalbumin (OVA, Sigma-Aldrich) every other day for 12 days before sampling as described previously [18]. In the SHOVA group, mice underwent the same OVA treatment regimen for 12 days, followed by a 4-week period of spontaneous healing before sampling. Mice were euthanized by cervical dislocation under deep isoflurane anaesthesia and the back skin was immediately collected. The protocol aimed to model acute allergic dermatitis (OVA group), spontaneous resolution (SHOVA group), and baseline skin status (CON group), enabling comparisons of inflammatory and healing responses. The immune organ index was calculated as the ratio of organ weight to body weight. Skin pH was measured using a pH detector (SMART SENSOR, pH828M), while skin moisture content and elasticity scores were assessed using a moisture analyzer (Real Bubee, RBX-916). No inclusion or exclusion criteria were established a priori. All animals and data points from each group were included in the experiment and subsequent analyses without any exclusions. Group allocation and experimental procedures were conducted by investigators who were aware of group assignments. However, outcome assessment and data analysis were performed by independent researchers blinded to the group allocation to minimize bias. To minimize potential confounders, treatment and measurement orders were randomized, and animals were housed with balanced cage placement.

### Data collection and processing

The single-cell sequencing data for human skin tissue in this study are available in the NCBI Gene Expression Omnibus (GEO) under accession numbers GSE153760 and GSE162054. The single-cell transcriptomic data for mouse skin are deposited under accession code GSE194254. The mouse dataset was not integrated with the human datasets; instead, all analyses were performed within species, and cross-species comparisons were limited to biological and functional interpretation. Ethical approval for the use of these datasets was obtained from the Ethics Committee of Beijing Obstetrics and Gynecology Hospital, Capital Medical University. The committee granted an exemption for the use of anonymized data (Exemption No. 2025-MS-001-01). This study complies with the Declaration of Helsinki. Following standardized scRNA-seq workflows [20], low-quality cells and doublets were filtered out using Seurat package (v4.3.0) and DoubletFinder package (v2.0.4) [21]. To correct for batch effects, we applied Seurat’s standard SCT-based integration workflow. Integration anchors were identified across samples using FindIntegrationAnchors (normalization.method = ‘SCT’), followed by dataset integration with IntegrateData [20,22]. This approach effectively reduces batch effects while preserving biological variability. Following integration, downstream analysis included data scaling, PCA, and UMAP for dimensionality reduction and visualization [23], and cluster-specific marker genes were identified using Seurat’s differential expression analysis.

### Construction of cell pseudotime trajectory

Using the ‘subset’ function, we extracted the keratinocyte population identified by Seurat analysis, and subsequently performed pseudotime trajectory analysis on both mouse and human keratinocytes using the Monocle package (v2.26) [24]. Briefly, high-variance genes were selected using the ‘FindVariableFeatures’ function, and a ‘CellDataSet’ was constructed. The data were then normalized and dispersion was estimated using the ‘estimateSizeFactors’ and ‘estimateDispersions’ functions. Next, genes with high dispersion were selected as ordering genes using the ‘dispersionTable’ and ‘setOrderingFilter’ functions. Dimensionality reduction was performed with the ‘reduceDimension’ function, and pseudotime trajectory was inferred using the ‘orderCells’ function. To further explore gene expression changes along the pseudotime trajectory, the BEAM function was applied to identify genes associated with different cell states, and a branched heatmap was generated using the ‘plot_genes_branched_heatmap’ function to visualize dynamic gene expression patterns.

### Differentially expressed genes (DEGs) identification and functional analysis

After normalizing the data, differential gene expression between groups was assessed using the ‘FindMarkers’ function from the Seurat package. DEGs were defined by a log2 fold change greater than 0.25 (absolute value) and a Benjamini-Hochberg false discovery rate (FDR)-adjusted p-value below 0.05. Additionally, branch-specific high-expression gene sets were identified using the Monocle package. These DEGs were further subjected to functional enrichment analysis.

For functional enrichment, we utilized the ‘clusterProfiler’ package (v4.6.2) to perform Gene Ontology (GO) biological process and Kyoto Encyclopedia of Genes and Genomes (KEGG) pathway analysis [25]. Significant pathways were identified based on adjusted p-values less than 0.05. The results of the functional enrichment analysis were visualized using the ggplot2 package (v3.5.1), with bar plots or bubble plots employed to highlight the most enriched biological pathways.

### Cell-cell communication analysis

The CellChat packages (v1.6.1) were used for ligand-receptor pair analysis [26]. The scRNA-seq data from the Seurat object were used to extract each group, filtered based on cell type annotations. A CellChat object was created using the scRNA-seq count data and cell type information. The analysis utilized the CellChatDB (mouse or human) database, focusing on ‘Secreted Signaling’ pathways. Overexpressed genes and interactions were identified, followed by the projected onto a mouse protein-protein interaction network. The cell-cell communication probabilities were computed, excluding interactions with insufficient cell numbers. The communication networks were inferred at the signalling pathway level, aggregated, and centrality scores computed and visualized as heatmaps. The analysis was extended to compare communication interactions and strengths between groups. The final results were visualized using chord diagrams, bubble plots, and 2D scatter plots to illustrate significant intercellular interactions and signalling roles between groups.

### Immunohistochemistry

Mouse skin tissue was fixed overnight in paraformaldehyde. After fixation, tissues were dehydrated through a graded alcohol series and cleared with xylene, followed by embedding in paraffin wax at 65 °C. The embedded tissues were then cooled, sectioned at 4 μm thickness, and mounted on slides. After drying at 60 °C, paraffin sections were stored at room temperature until use.

The paraffin sections were dewaxed and rehydrated through graded ethanol solutions. Antigen retrieval was performed, and the sections were washed with PBS. Endogenous peroxidase activity was blocked with 3% hydrogen peroxide. The sections were then blocked with 3% BSA or serum and incubated with primary antibodies overnight at 4 °C. After washing, the secondary antibodies (HRP-labelled) was applied, and DAB colour development was performed, with positive staining visualized as brown or yellow signals. The sections were restained with haematoxylin, dehydrated, and mounted with a sealing medium. Finally, images were captured using a microscope (Nikon, Tokyo, Japan). Primary antibodies used included CD11c (DF7585, Affinity Biosciences) to label dendritic cells, CK-14 (60320-1, Proteintech) for keratinocytes, CD3 (17617-1, Proteintech) as a marker for T lymphocytes, and F4/80 (28463-1, Proteintech) to identify macrophages. Positive cells were quantified by counting multiple non-overlapping 200 μm segments along the basal layer of the epidermis.

### RNA extraction and real-time quantitative PCR (RT-qPCR)

Total RNA was extracted from skin tissues using the FastPure Complex Tissue/Cell Total RNA Isolation Kit (RC113-01, Vazyme), following the manufacturer’s protocol. cDNA was synthesized using the HiScript III 1st Strand cDNA Synthesis Kit (R312-02, Vazyme) according to the provided instructions. Real-time quantitative PCR (RT-qPCR) ChamQ Universal SYBR qPCR Master Mix (Q711-02, Vazyme) was performed using the LC480 real-time PCR system (Bio-Rad, California, USA) with the specified PCR conditions. The primers used are listed in Table S1. mRNA expression levels were quantified using the 2^−△△Ct method.

### Statistical analysis

Phenotypic data and RT-qPCR results were analyzed and presented as mean ± standard deviation (SD) using GraphPad Prism 8 software. Statistical significance between two groups was assessed using an unpaired two-tailed Student’s t-test, with effect sizes (Cohen’s d) calculated. For comparing three groups, one-way ANOVA followed by Tukey’s post-hoc test was performed. Asterisk symbols indicate statistical significance (*p < 0.05, **p < 0.01), and effect sizes (η^2^) were calculated. The non-significant differences are indicated as n.s. (*p* > 0.05). For bioinformatics analyses, R software (v4.2.1) was used for data visualization and statistical analysis following standard protocols.

## Results

### Increased immune cell infiltration and activation of inflammatory pathways in AD

**Figure 1.**
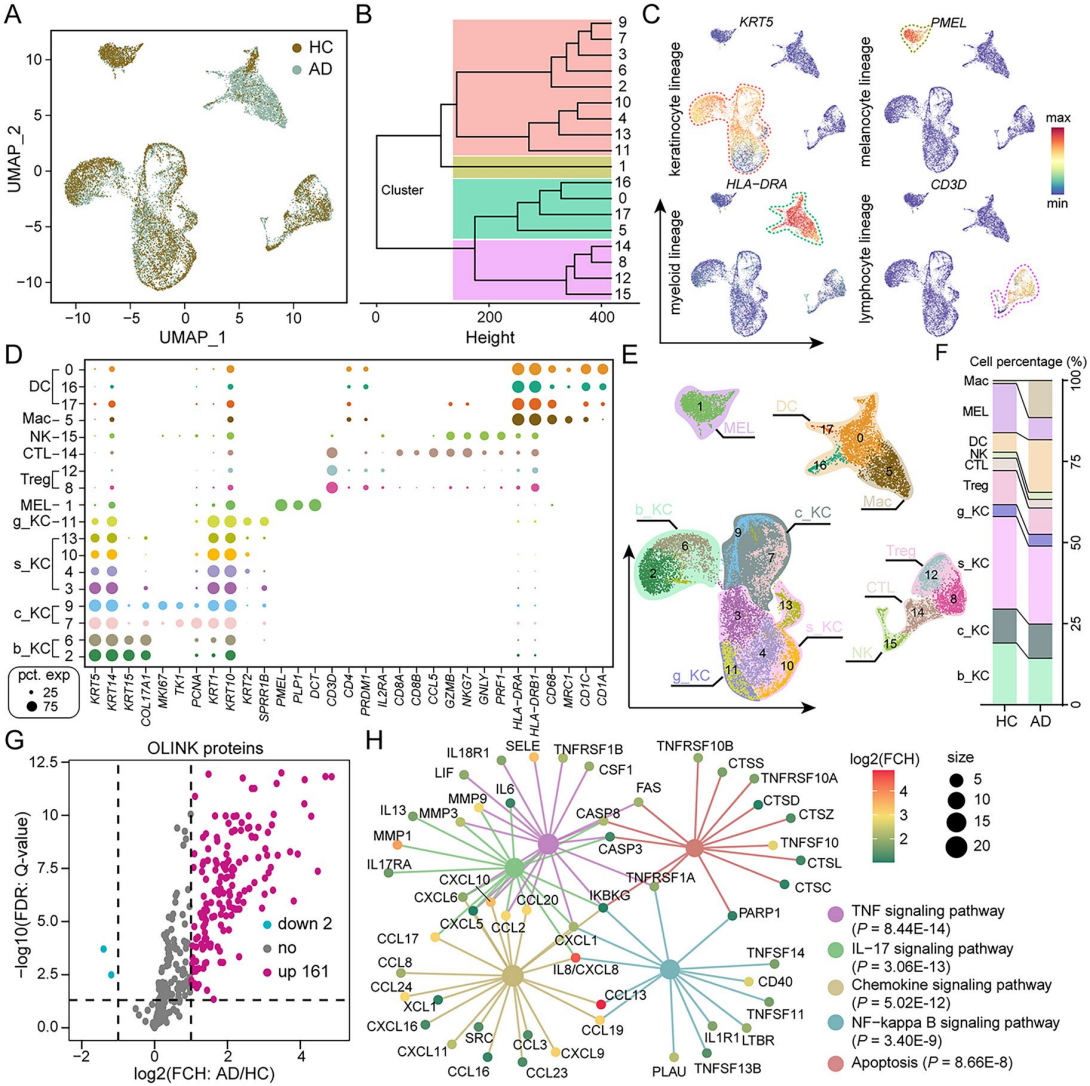
Single-cell transcriptome and proteomic profiling of healthy control (HC) and atopic dermatitis (AD) skin samples. (A) Uniform manifold approximation and projection (UMAP) plot colour coded by cell source in each group. (B) Hierarchical clustering of different cell clusters identified by UMAP analysis. (C) Feature plots of expression distribution for exemplary cluster-specific marker genes. (D) Representative canonical cell type-specific marker expression across all cell clusters. (E) UMAP projection of single-cell profiles reveals 18 cell clusters and 10 cell types. (F) The percentages of cell types from different groups. (G) Volcano plots of proteins detected by proteomic multiplex assays in HC and AD groups. (H) The KEGG pathway analysis of differential proteins highlighted five significantly enriched key pathways. Mac: Macrophages; DC: Dendritic Cells; NK: Natural killer cells; CTL: Cytotoxic T lymphocytes; Treg: Regulatory T lymphocytes; MEL: Melanocytes; b_KC: Basal keratinocytes; c_KC: Cycling keratinocytes; g_KC: Granular keratinocytes; s_KC: Spinous keratinocytes.

To investigate the potential mechanisms underlying keratinocytes in AD, we performed an in-depth analysis of previously generated single-cell transcriptomic data from healthy controls (HC) and AD patients, obtained *via* skin suction blistering [27]. As outlined in Figure S1A, scRNA-seq was performed on skin tissues after enzymatic digestion and flow cytometric sorting of CD45-positive and CD45-negative cells, which were mixed in equal proportions to ensure sufficient immune cell representation. After filtering out low-quality and doublet cells, we obtained a total of 18,239 high-quality cells and performed dimensionality reduction analysis (Figure 1A and Figure S1B-S1D). These cells were categorized into 18 clusters (0–17). Unsupervised clustering further revealed that they could be grouped into four major cell populations (Figure 1B). Marker gene analysis identified these populations as the *KRT5*-expressing keratinocyte lineage, *PMEL*-positive melanocyte lineage, *HLA-DRA*-positive myeloid lineage, and *CD3D*-positive lymphocyte lineage (Figure 1C). The method of defining cell populations using specific markers has been applied in previous studies [16,27,28]. Following this approach, we classified the cells into 10 groups based on the markers, and as an example, in the keratinocyte lineage, we identified four subtypes: cycling keratinocytes (c_KC) expressing *MKI67*, *TK1*, and *PCNA* [27]; granular keratinocytes (g_KC) expressing *KRT2* and *SPRR1B* [29]; basal keratinocytes (b_KC) expressing *KRT15* and *COL17A1* [30]; and spinous keratinocytes (s_KC) expressing *KRT10* and *KRT1* [31] (Figure 1D, 1E, and Figure S1E). We observed a significant increase in the proportion of immune cells in AD, indicating the occurrence of immune cell infiltration (Figure 1F). To further investigate the impact of infiltrating immune cells on the tissue microenvironment, we analyzed previously published proteomic data [6], which revealed a significant upregulation of various inflammatory markers (Figure 1G). KEGG pathway analysis indicated that these upregulated proteins were primarily enriched in the ‘TNF signaling pathway’, ‘IL-17 signaling pathway’, ‘chemokine signaling pathway’, ‘NF-κB signaling pathway’, and ‘apoptosis’ (Figure 1H). In conclusion, our results demonstrate a significant increase in immune cell infiltration in the skin of AD patients, accompanied by notable upregulation of key inflammatory pathways.

### Activation of antigen processing and presentation in keratinocytes of AD

**Figure 2.**
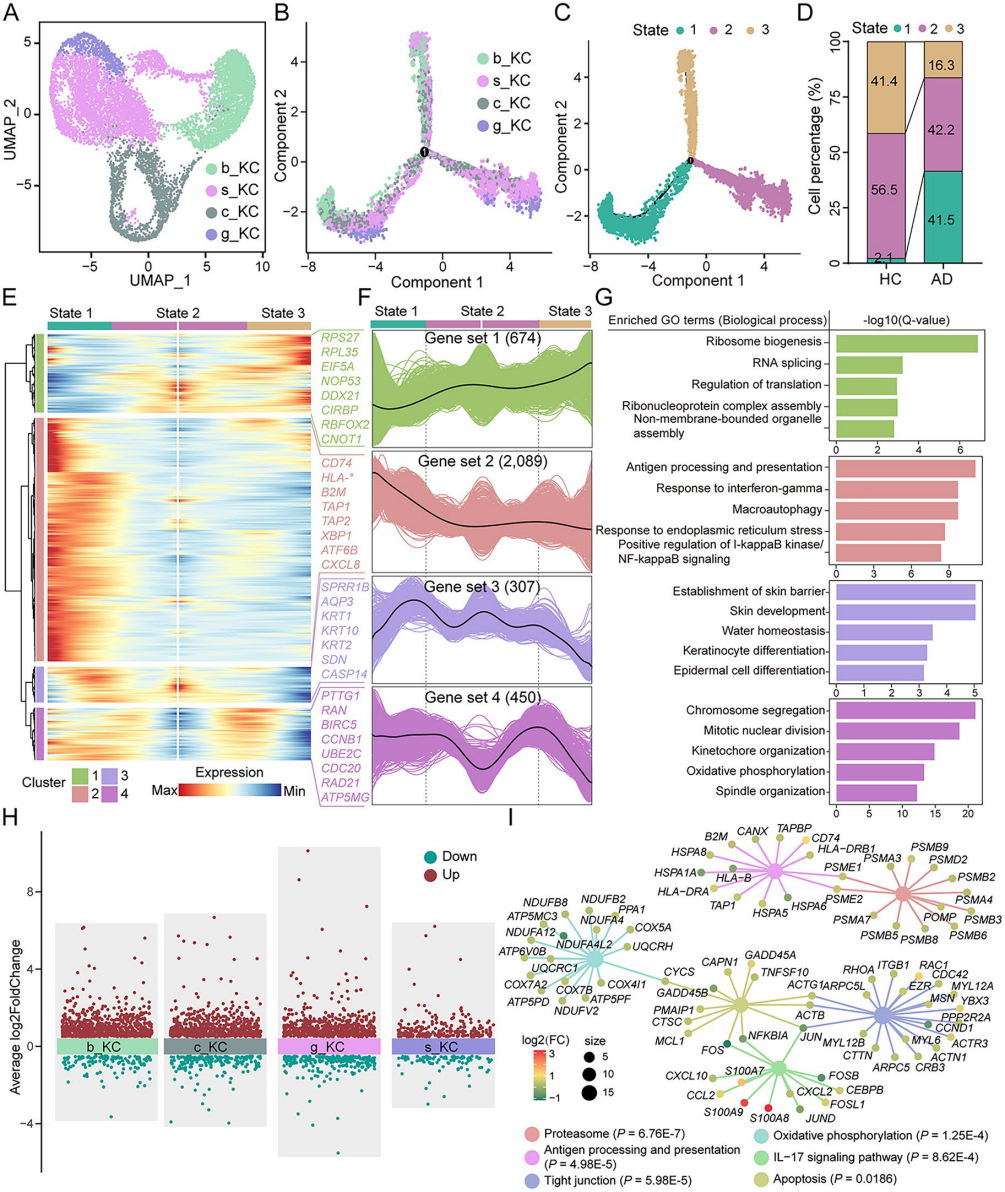
Keratinocyte cell fate and transcriptional profiles are significantly altered in AD. (A) Subpopulation of keratinocyte clusters coloured based on four cell subtypes. (B) Single-cell pseudotime developmental trajectory of keratinocyte populations coloured by four cell subtypes, which were identified with two developmental fates. (C) Single-cell pseudotime developmental trajectory of keratinocyte populations coloured by three states. (D) Cell percentages of three states among two sample groups. (E) Pseudotime ordered heatmap of four differentially expressed gene (DEG) sets between two obvious fates at branch point one. (F) The graph shows the expression of the four DEG sets in the pseudotime trajectory. (G) The enrichment of GO terms in each gene set. (H) Volcano plots of differential analysis between HC and AD based on four cell subpopulations. (I) The KEGG enrichment analysis of DEGs in keratinocytes between the two groups highlighted five significantly enriched key pathways.

Keratinocytes, as the primary barrier of the skin, account for over 90% of skin cells. However, previous studies have rarely provided an in-depth analysis of their potential driving role in AD. In this study, we performed pseudotime analysis on extracted keratinocytes, which revealed three distinct transcriptional states and two main branching fates (Figure 2A-2C and Figure S2A). Statistical analysis further demonstrated that, compared to HC, the proportion of keratinocytes in state 1 was significantly increased in AD, while the proportion of cells in state 3 was markedly reduced (Figure 2D).

Based on the gene expression patterns of two branches, the study identified four gene sets associated with two distinct cellular fates, whose expression significantly fluctuates along the cell trajectory (Figure 2E and 2F). Specifically, the representative genes of state 1 correspond to 2,089 genes in gene set 2, which are closely associated with immune functions such as ‘antigen processing and presentation’, ‘response to interferon-gamma’, ‘macroautophagy’, ‘endoplasmic reticulum stress response’, and ‘positive regulation of I-kappaB kinase/NF-kappaB signaling’ (Figure 2E-2G). These findings may underlie the core mechanisms contributing to AD. In contrast, the representative genes of state 3 are associated with 674 genes in gene set 1, which are involved in transcription-related processes, including ‘ribosome biogenesis’, ‘RNA splicing’, and ‘regulation of translation’ (Figure 2E-2G). Additionally, the representative genes of state2 correspond to 307 genes in gene set 3, which are linked to barrier functions, such as ‘establishment of skin barrier’, ‘skin development’, and ‘water homeostasis’ (Figure 2E-2G).

To further investigate whether these transcriptional changes are concentrated in specific subsets of keratinocytes, we assessed the distribution of cells across different states. Consistent with previous findings [30], we observed an increase in the proportion of c_KC and a decrease in b_KC in the AD group (Figure S2B). Notably, four distinct keratinocyte subtypes were identified within the significantly increased state 1 in the AD group (Figure S2B). Differential analysis of these subtypes revealed that the number of shared genes was greatest among them (Figure 2H and Figure S2C). These findings indicate that transcriptional reprogramming in keratinocytes during AD may represent a broad phenomenon influencing the keratinocyte across the lineage, rather than being restricted to a specific cell subset. Subsequently, we performed differential gene expression analysis and functional enrichment on keratinocytes (Figure 2I and Figure S2D, S2E). Notably, KEGG pathway analysis revealed significant enrichment of several pathways associated with AD, including the ‘proteasome’, ‘oxidative phosphorylation’, ‘tight junctions’, ‘IL-17 signaling pathways’, and ‘apoptosis’ (Figure 2I). Interestingly, the pathway for ‘antigen processing and presentation’ was also significantly enriched among these pathways (Figure 2I). Considering the crucial role of this pathway in regulating immune responses, it is speculated that its involvement may contribute to the chronic, persistent inflammation characteristic of AD.

### Altered intercellular communication between keratinocytes and immune cells in AD

**Figure 3.**
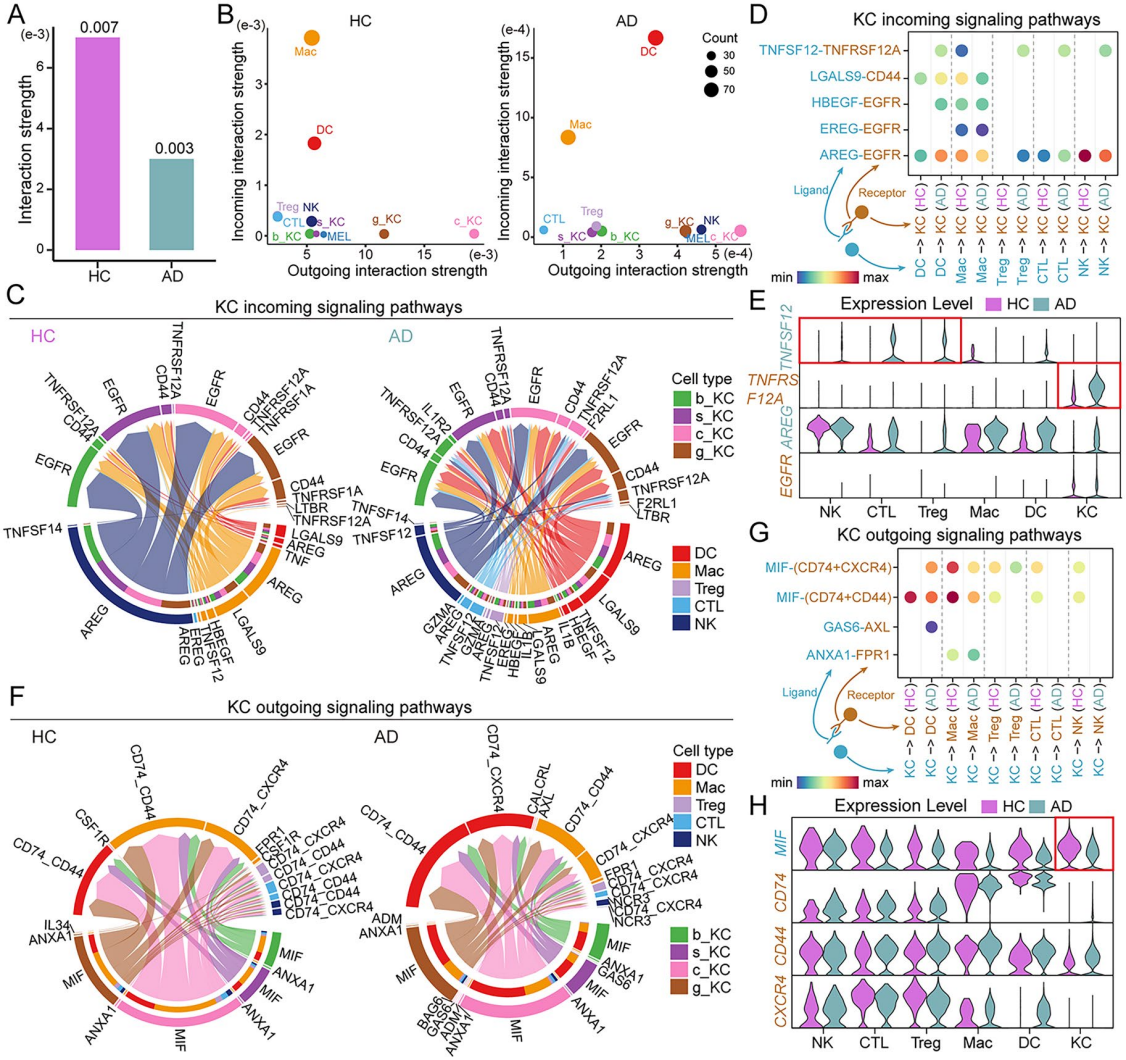
AD interrupts communication between keratinocytes and immune cells. (A) Bar chart showing interaction strength in skin of HC and AD groups. (B) 2D spatial maps of the intensity of incoming interaction strength and outgoing interaction strength of 10 major cell groups in skin of HC and AD groups. (C) Chord charts illustrate the expression levels of incoming signaling pathways from five immune cell types to four keratinocyte subtypes in the HC and AD groups. (D-E) The dot plot and violin plots show the expression levels of differentially regulated ligand-receptor pairs from five immune cell types to keratinocytes in two groups. (F) Chord charts illustrate the expression levels of outcoming signaling pathways from four keratinocyte subtypes to five immune cell types in the HC and AD groups. (G-H) The dot plot and violin plots show the expression levels of differentially regulated ligand-receptor pairs from keratinocytes to five immune cell types in two groups.

To further investigate the regulatory role of keratinocytes on immune cells, we performed an in-depth analysis of intercellular communication. CellChat analysis revealed a significant decrease in the inferred interaction strength within the cell communication network in the AD group compared to the HC group (Figure 3A), with AD causing alterations in the input and output signalling patterns between cells to varying degrees (Figure 3B). We then extracted and focused on the ligand-receptor pairs between the four keratinocyte subsets and various immune cells. The results indicated that, despite differences in communication patterns, the signal reception profiles of the four keratinocyte subtypes were largely overlapping (Figure 3C). This finding supports the concept of widespread transcriptional reprogramming in AD keratinocytes. Therefore, we analyzed the immune signal reception by keratinocytes as a whole in AD. The results revealed that the TNFSF12-TNFRSF12A pathway was significantly activated in AD, which could be a key factor in keratinocyte apoptosis (Figure 3D and 3E). Meanwhile, the output signalling of the four keratinocyte subtypes exhibited similar ligand-receptor patterns (Figure 3F). Analysis of keratinocyte output signals revealed that, compared to the HC group, the MIF-CD74/CD44/CXCR4 signalling pathway was significantly suppressed in the AD group (Figure 3G and 3H). These results indicate that in AD, the intercellular communication between keratinocytes and immune cells is altered.

### Transcriptional changes in keratinocytes of antigen-driven AD mouse models differ from those in human AD

**Figure 4.**
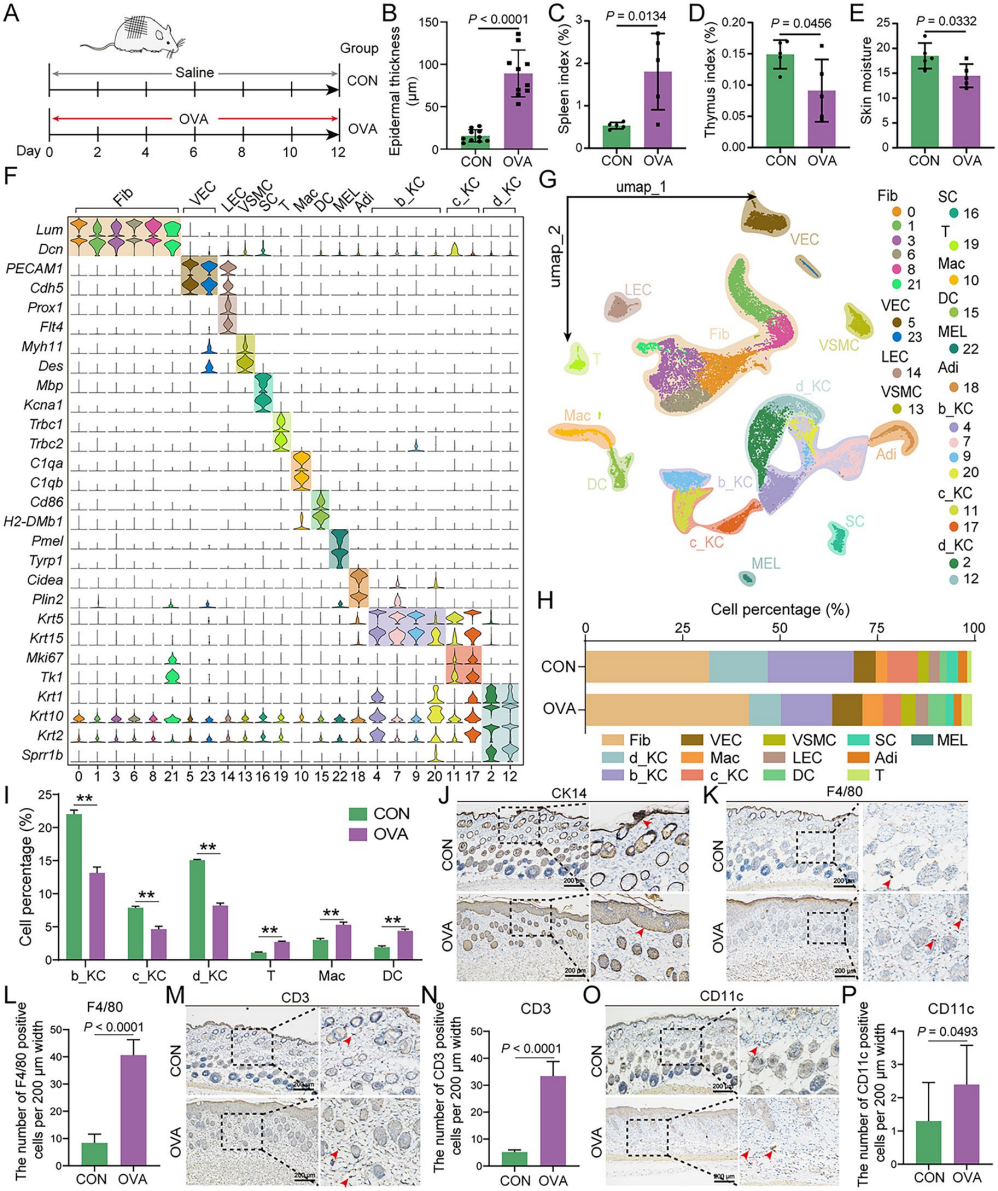
Single-cell transcriptome and phenotype profiling of control (CON) and ovalbumin (OVA)-induced AD mouse model groups. (A) Schematic diagram illustrating the induction model for CON group and OVA group in mice. (B) The bar plot shows the comparison of epidermal thickness between the control and OVA-treated groups. (C-D) The bar plot shows the comparison of spleen index and thymus index between the control and OVA-treated groups. (E) The bar plot shows the comparison of skin moisture between the control and OVA-treated groups. (F) Violin plots of expression distribution for exemplary cluster-specific marker genes. (G) UMAP projection of single-cell profiles reveals 24 cell clusters and 13 cell types. (H) The percentages of cell types from different groups. (I) Bar plot shows the distribution of six selected cell populations in two groups. (J) Representative IHC images of CK14 in the skin of CON and OVA groups. Scale bar, 200 μm. (K-P) Representative IHC images and corresponding statistical bar graphs of F4/80, CD3, and CD11c in the skin of CON and OVA groups are shown. Scale bar, 200 μm. Fib: Fibroblasts; VEC: Vascular endothelial cells; LEC: Lymphatic endothelial cells; VSMC: Vascular smooth muscle cells; SC: Schwann cells; T: T lymphocytes; Mac: Macrophages; DC: Dendritic cells; MEL: Melanocytes; Adi, Adipocytes; b_KC: Basal keratinocytes; c_KC: Cycling keratinocytes; d_KC: Differentiating keratinocytes.

Although keratinocyte antigen processing and presentation can drive inflammatory responses, it remains unclear whether allergens directly induce this aberrant activity. To investigate this issue, we established an OVA-induced AD mouse model following previous protocols (Figure 4A) [18]. Compared with the control group (CON), OVA-induced mice exhibited epidermal thickening, significant changes in immune organ indices (increased spleen index and decreased thymus index), and markedly reduced skin moisture, while skin elasticity score and pH remained unaffected (Figure 4B–4E and Figure S3A-S3F). These phenotypic changes recapitulate key features of human AD. Subsequently, we analyzed the previously collected single-cell transcriptomic data from the control (CON) and OVA-treated mouse skin samples [18]. After filtering out low-quality cells and performing dimensionality reduction and clustering, we identified 23 distinct cell clusters (labelled 0–22) (Figure 4F and Figure S4A-S4C). Using known marker genes and highly expressed genes, these clusters were classified into 13 cell types, including three types of keratinocytes and three types of immune cells (Figure 4G and Figure S4D). Notably, in the OVA group, the proportion of immune cells was significantly increased, whereas the proportion of keratinocytes was markedly reduced (Figure 4H and 4I). Furthermore, immunohistochemical staining reduced CK14 staining intensity in OVA skin, together with a marked increase in the number of F4/80, CD3, and CD11c-positive cells (Figure 4J-4P). F4/80, CD3 and CD11c predominantly mark macrophages (Mac), T lymphocytes (T) and dendritic cells (DC), respectively. These results suggest that in the OVA-induced AD mouse model, there is a significant alteration in the skin cell composition, with a marked increase in immune cell infiltration.

**Figure 5.**
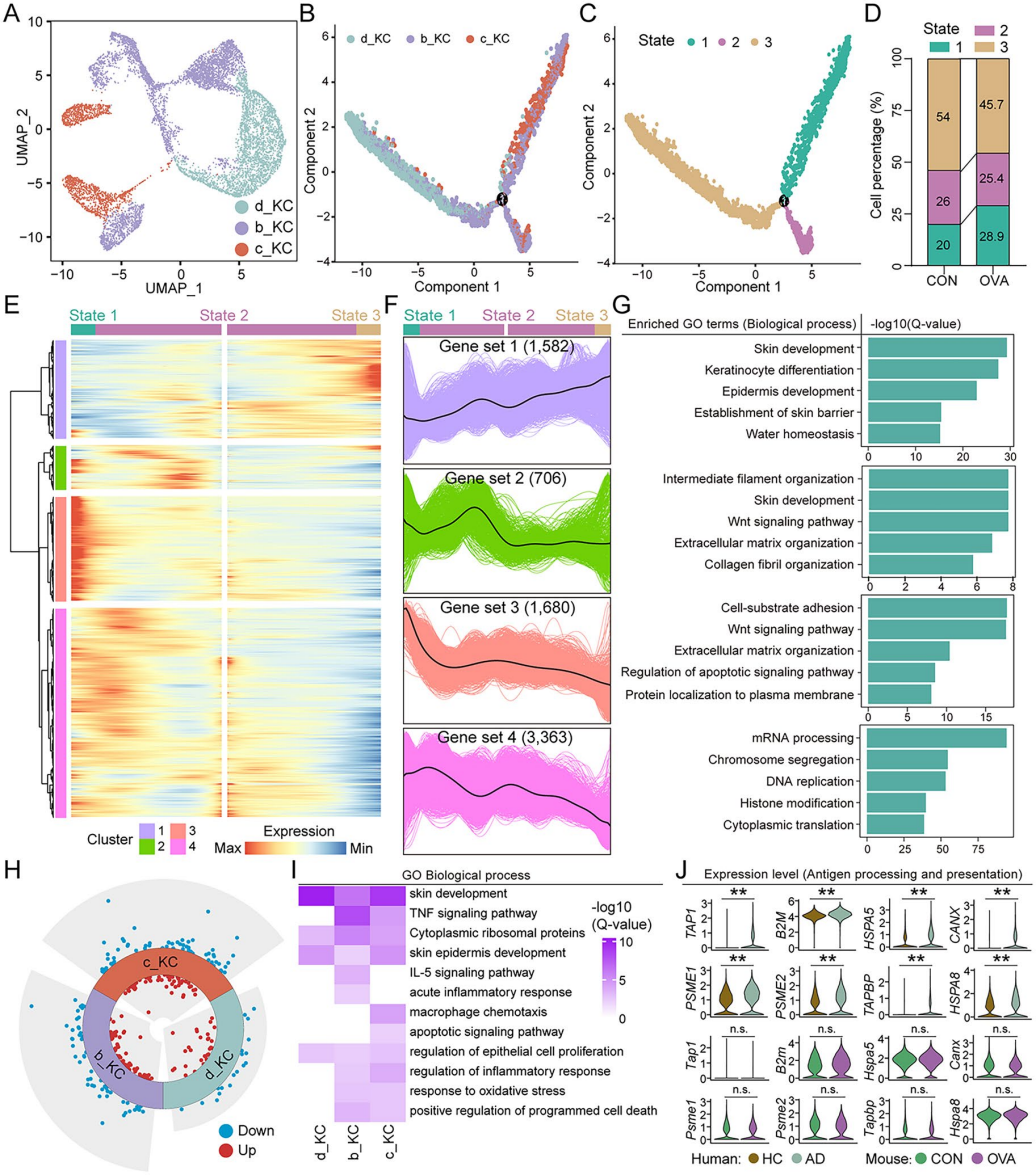
Keratinocyte cell fate and transcriptional profiles are significantly altered in the OVA group. (A) Subpopulation of keratinocyte clusters coloured based on three cell subtypes. (B) Single-cell pseudotime developmental trajectory of keratinocyte populations coloured by three cell subtypes, which were identified with two developmental fates. (C) Single-cell pseudotime developmental trajectory of keratinocyte populations coloured by three states. (D) Cell percentages of three states among CON and OVA groups. (E) Pseudotime ordered heatmap of four DEG sets between two obvious fates at branch point one. (F) The graph shows the expression of the four DEG sets in the pseudotime trajectory. (G) The enrichment of GO terms in each gene set. (H) Volcano plots of differential analysis between CON and OVA based on three cell subpopulations. (I) Heatmap demonstrates the GO enrichment results of DEGs in three cell subpopulations. (J) The expression of antigen processing and presentation-related genes in human (HC, AD) and mouse models (CON, OVA). n.s.: not significant; **p < 0.01.

We further conducted a pseudotime analysis of keratinocytes from CON and OVA mice to determine whether there were similar alterations to those observed in human AD. Our analysis revealed that the keratinocytes from the mouse models were divided into three distinct cell states, akin to the human results (Figure 5A-5C). Compared to the CON group, the OVA group exhibited an increase in state 1 and a decrease in state 3 (Figure 5D). We then identified genes whose expression varied significantly along the two trajectory branches. We found that 1,680 genes (Gene set 3) highly expressed in state 1 were significantly enriched in pathways related to ‘cell-substrate adhesion’, ‘wnt signaling pathway’, ‘extracellular matrix organization’, ‘regulation of apoptotic signaling pathway’, and ‘protein localization to plasma membrane’ (Figure 5E-5G). In contrast, 1,582 genes (Gene set 1) highly expressed in state 3 were primarily associated with functions such as ‘skin development’, ‘keratinocyte differentiation’, ‘epidermis development’, ‘establishment of skin barrier’, and ‘water homeostasis’ (Figure 5E-5G). Moreover, both in analyses of the three keratinocytes subpopulations and in global comparisons, the number of DEGs was substantially lower than that observed between AD and HC groups (Figure 5H and Figure S5A). The functional enrichment of these DEGs was primarily associated with ‘skin development’ (Figure 5I and Figure S5B). Additionally, KEGG pathway analysis revealed significant enrichment in the ‘TNF signaling pathway’ and ‘IL-17 signaling pathway’ (Figure S5C). However, compared to the CON group, we did not observe any significant changes in genes related to antigen processing and presentation in the OVA group (Figure 5J). The significantly altered ligand-receptor signalling pathways in the OVA group were primarily influenced by changes in immune cells, rather than keratinocytes (Figure S5D and S5E). Comparison with human AD revealed notable discrepancies, particularly in keratinocyte transcriptomic reprogramming and antigen-presentation signatures, suggesting limited concordance between the OVA model and human disease.

### Increased immune cell infiltration and transcriptional reprogramming of keratinocytes in SHAD

**Figure 6.**
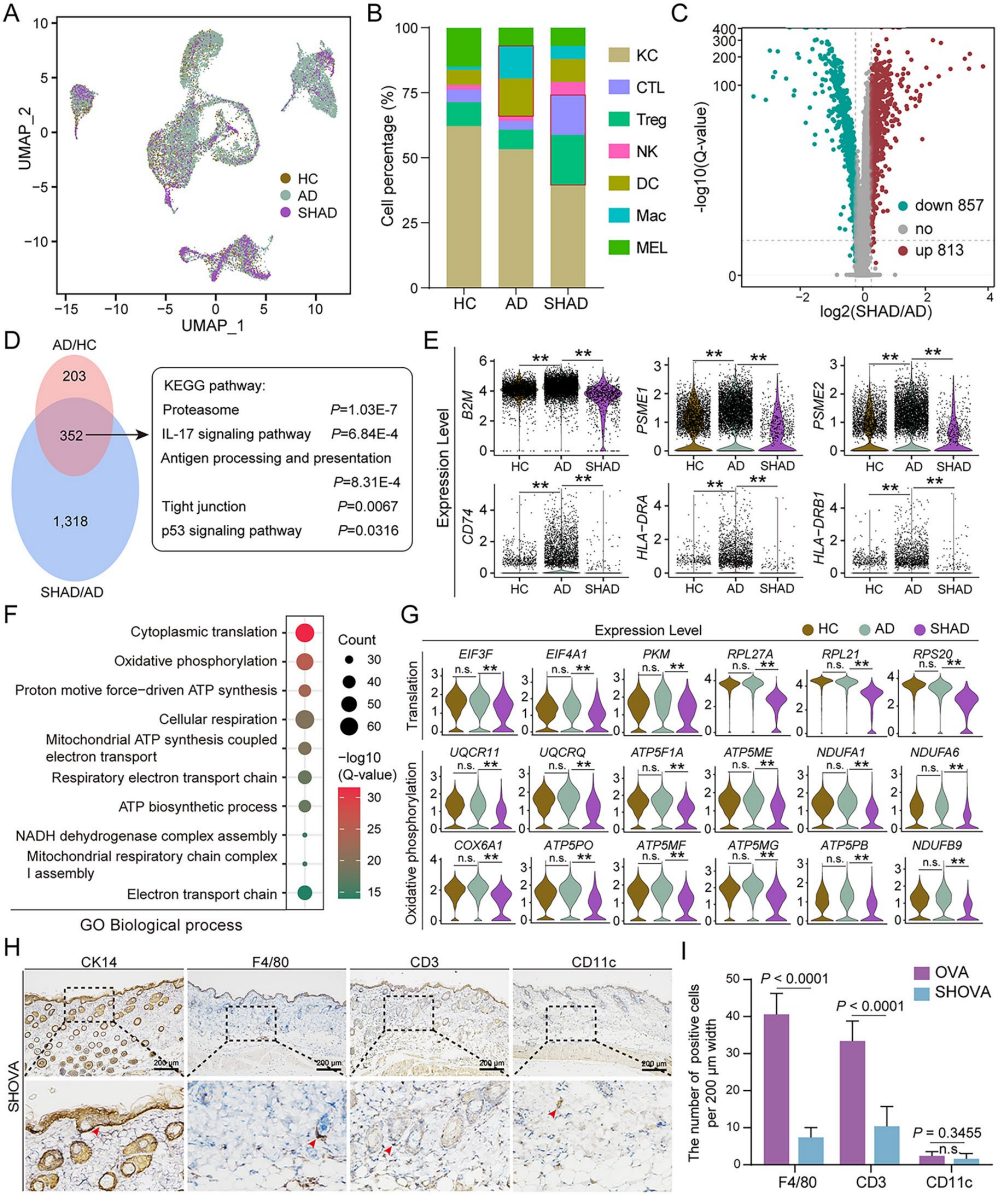
Single-cell transcriptome profiling of HC, AD, and spontaneously healed AD (SHAD) skin samples. (A) UMAP plot colour coded by cell source in HC, AD, and SHAD groups. (B) The percentages of cell types from different groups. (C) The volcano plot showing the DEGs of keratinocyte between AD and SHAD groups. (D) Venn diagram shows the DEGs of keratinocytes between AD and SHAD groups (left) and the GO enrichment results (right). (E) The expression of antigen processing and presentation-related genes in HC, AD, and SHAD groups. (F) The bubble chart shows the GO (biological process) enrichment results of DEGs between AD and SHAD groups. (G) The expression of cytoplasmic transcription and oxidative phosphorylation-related genes in HC, AD, and SHAD groups. (H) Representative IHC images of CK14, F4/80, CD3, and CD11c in the skin of CON and OVA groups are shown. Scale bar, 200 μm. (I) Bar graphs of positive cells for F4/80, CD3, and CD11c in the skin of CON and OVA groups are shown. Scale bar, 200 μm. n.s.: not significant; **p < 0.01.

Some patients with AD exhibit a self-resolving pathological process after a certain period. Investigating the tissue-specific single-cell characteristics during this self-resolving process could provide valuable insights into the mechanisms underlying AD treatment. To this end, we integrated previously obtained single-cell samples [32] from SHAD and applied the clustering and integration methods used in earlier studies. This resulted in the identification of 10 distinct cell subsets (Figure 6A, Figure S6A-S6C). Cell proportion analysis revealed that the immune cell composition in the SHAD group did not return to the levels observed in HC; instead, the immune cell proportions continued to increase (Figure 6B). Notably, among the immune cells in AD patients, myeloid-derived DC and Mac were predominant, whereas in the SHAD group, Tregs and CTL were the most abundant (Figure 6B). This finding prompted further investigation into the changes in keratinocytes. Differential gene expression analysis of keratinocytes revealed significant transcriptional changes in the SHAD group, with 813 genes upregulated and 857 genes downregulated (Figure 6C). The extent of these changes surpassed the differences observed between the AD and HC groups. Further comparison of these differential genes showed that the significantly restored genes were primarily enriched in biological pathways associated with AD pathogenesis (Figure 6D), particularly those related to antigen processing and presentation (Figure 6E).

A subsequent analysis of 1,318 genes that exhibited specific and significant changes in the SHAD group revealed that these genes were mainly involved in cytoplasmic transcription and oxidative phosphorylation processes (Figure 6F). Notably, the downregulated genes occupied central positions in pathway enrichment analysis (Figure 6G), while the upregulated genes were primarily associated with mRNA metabolism (Figure S6D). Cellular communication analysis revealed that receptor-mediated signalling pathways were significantly altered in AD compared to the control, but showed clear recover in the SHAD group (Figure S6E and S6F). These findings suggest that the changes in the transcriptional profile of keratinocytes in the SHAD group may reflect an adaptive response to alterations in immune cell composition.

After the withdrawal of the allergen in the OVA-induced mouse model of allergic dermatitis, followed by a 4-week self-healing period, we observed that the skin phenotype had returned to control levels (Figure S7A and S7B). Subsequent analyses revealed that the relevant indices had recovered to varying extents (Figure S7C-S7G). Further histological staining showed a significant reduction in immune cell numbers in the skin of the SHOVA group, with the exception of DC, which exhibited a decreasing trend, although the decrease was not statistically significant (Figure 6H and 6I). Additionally, no downregulation of oxidative phosphorylation and transcriptional levels was observed in the SHAD group (Figure S7H). These results suggest that the self-healing process in AD is relatively complex, likely involving multiple cellular balance adjustments. A more suitable mouse model is still needed to fully analyze this disease course. In summary, our findings highlight the dynamic adjustments of keratinocytes in the immune microenvironment during the self-resolving process of AD. These adjustments likely play a crucial role in the self-healing mechanisms of the disease.

## Discussion

In recent years, significant advancements have been made in understanding the role of immunological responses and inflammatory mediators in AD [33–35]. However, the precise contributions of keratinocytes in AD, particularly in the context of immune responses, remain largely unresolved [12]. Research has demonstrated that keratinocytes serve as both the first line of defence against initial pathological insults and key mediators that exacerbate disease pathogenesis [36]. In inflammatory conditions, cytokines and growth factors such as IL-1β, TNF-α, IFN-γ, and others can stimulate keratinocyte activation [37]. Activated keratinocytes, in turn, release chemokines like CCL5 and CXCL10, which further trigger immune cell cascades, enhancing skin sensitivity to allergens and contributing to the onset of pruritus [38,39]. The results of this study indicate a significant increase in the levels of numerous inflammation-associated factors in AD (Figure 1H). With the advent of single-cell omics technologies, emerging evidence suggests that keratinocytes play a much more active and central role in the pathogenesis of AD than previously appreciated [30]. A recent scRNA-seq study on AD [30], consistent with our findings, revealed altered keratinocyte differentiation (Figure 2). In contrast, our study did not provide strong evidence for antigen processing and presentation being restricted to particular keratinocyte subpopulations, but rather pointed towards a more global reorganization of the keratinocyte transcriptional landscape. The broadly similar ligand-receptor patterns observed across the four keratinocyte subtypes may further reflect this trend.

In AD patients, keratinocytes exhibit a marked upregulation of genes related to antigen processing and presentation, indicating their active role in modulating immune responses in the epidermis. Although keratinocytes’ ability to process and present antigens is well recognized [40,41], it has traditionally been viewed as a secondary function, assisting antigen-presenting cells in modulating immune responses. However, our findings suggest a potentially more prominent immunoregulatory role of keratinocytes in AD than previously recognized. We observed significant alterations in the communication between keratinocytes exhibiting activated antigen processing and presentation functions and immune cells in AD. However, in the SHAD group, this disrupted communication was restored as the antigen processing and presentation functions of keratinocytes diminished, with keratinocyte changes appearing to play a central role in this process (Figure 3). By actively contributing to antigen presentation and immune modulation, keratinocytes may function as critical contributors in the immunopathogenesis of AD, influencing both the initiation and persistence of inflammation [42,43].

Although we did not perform direct functional experiments such as *in vitro* knockdown of HLA-DRA or *in vivo* MHC-II inhibition, several studies provide supporting evidence for the functional capability of keratinocytes to present antigens and activate T cells. For instance, Zima et al. showed that IFN-γ and proinflammatory cytokines induce HLA-DRα and CD74 in human keratinocytes, promoting antigen processing and T cell activation, suggesting an important role in immune-mediated skin diseases [43]. Black et al. showed that IFN-γ–stimulated keratinocytes can activate CD4^+^ and CD8^+^ memory T cells, inducing cytokine production and cytotoxic responses [44]. Fan et al. reported that keratinocyte-specific MHC-II expression in a TCR-transgenic mouse model drives CD4^+^ autoreactive T cell-mediated skin inflammation [45]. Together, these studies provide functional and *in vivo* evidence that supports our transcriptomic observations, highlighting the potential of keratinocytes as active contributors to chronic inflammation in AD. Future studies should employ functional assays, including cytokine secretion, antigen presentation, and *in vivo* keratinocyte-specific MHC-II perturbation, to directly validate the causal role of keratinocyte-mediated antigen presentation in AD pathogenesis.

In the OVA mouse model of allergic immune dysregulation, keratinocytes do not show significant activation of antigen presentation despite allergen exposure (Figure 5). This key finding suggests that, although previous studies have highlighted phenotypic parallels between this model and human AD [18], the OVA model may have limitations in recapitulating keratinocyte-mediated antigen presentation under the conditions examined in this study. The transcriptional alterations in keratinocytes from the OVA group are markedly less pronounced than those observed in keratinocytes derived from human AD (Figure S2D and S5A). Furthermore, the alterations in the OVA group are primarily driven by immune cells, whereas in human AD, the cellular communication networks are predominantly keratinocyte-driven, highlighting a significant disparity between the two conditions (Figure 3E, 3H and Figure S5E). Additionally, when we compared the SHOVA model to human SHAD, we observed starkly divergent patterns in both immune cell infiltration and keratinocyte transcriptomic changes (Figure 6). The recovery observed in AD may result from a more pronounced transcriptional reprogramming of keratinocytes in response to stronger immune cell infiltration. In contrast, following allergen withdrawal, the skin of SHOVA mice tends to revert to baseline levels, indicating that the OVA model does not fully capture the self-repair mechanisms and chronic immune response characteristic of AD (Figure 6). These discrepancies highlight the limitations of the OVA model in accurately modelling the complex pathophysiology of AD, particularly with respect to keratinocyte dysfunction and the prolonged immune response.

In eukaryotic cells, the primary source of energy is mitochondrial oxidative phosphorylation, which generates ATP through glucose metabolism to support critical cellular activities such as transcription [43,46]. Interestingly, the SHAD group maintains low levels of intracellular transcription and oxidative phosphorylation (Figure 6G). This downregulation of oxidative phosphorylation may represent an important feature associated with the self-healing process. By reducing mitochondrial electron transport chain activity, keratinocytes in SHAD tissue potentially lower reactive oxygen species (ROS) production, which are known secondary messengers that perpetuate inflammatory signalling and cellular damage [47,48]. Recent studies indicate that ROS modulate inflammatory responses in the skin, with altered ROS levels affecting AD pathology [49], and the KEAP1-NRF2 pathway is critical for redox balance in keratinocytes, where NRF2 activation can reduce oxidative stress and epidermal inflammation in AD [50]. A reduction in ROS is consistent with a less pro-oxidant environment, thereby mitigating oxidative stress and promoting a resolution phase that is conducive to skin tissue repair [51,52]. Alternatively, it may reflect a balanced adjustment of transcriptional activity in the keratinocytes during the self-healing process of AD. A key limitation of this study is the lack of suitable mouse models to accurately replicate the development of chronic AD in humans. In the near future, single-cell genomics will be essential to identify better models for studying keratinocyte behaviour.

Our findings raise the possibility that keratinocytes contribute to persistent immune activation *via* antigen presentation. Therefore, keratinocyte-directed interventions may complement current systemic therapies by targeting the disease-sustaining keratinocyte-T cell feedback loop in chronic atopic dermatitis. Current biologics (e.g. dupilumab, tralokinumab) and oral JAK inhibitors (e.g. abrocitinib) reliably dampen Th2-polarized inflammation and rapidly improve signs and symptoms, but they act largely downstream of keratinocyte antigen-processing pathways [53–56]. Preclinical and clinical evidence indicates that targeting keratinocyte-intrinsic signalling (such as JTE-052/delgocitinib) can restore differentiation and barrier proteins and blunt cytokine-driven transcriptional programs in keratinocytes [57,58]. Mechanistic support comes from the cytokine-induced upregulation of MHC class II on keratinocytes and the identification by single-cell studies of associated cellular states that link impaired healing to altered T-cell responses, underscoring their role in maintaining chronic inflammation [40,59]. Recent studies show that keratinocyte-targeted interventions can suppress nuclear translocation of pro-inflammatory factors, thereby protecting against inflammatory damage and potentially delaying AD progression [60]. Identifying interventions that modulate antigen processing and presentation in keratinocytes remains critical for effectively addressing AD pathogenesis. Taken together, combining systemic cytokine/JAK blockade with topical keratinocyte-targeted therapies represents a promising strategy to improve remission durability, though prospective trials with careful safety monitoring are warranted.

We acknowledge that the lack of longitudinal human data is a key limitation. While our cross-sectional analyses of AD and SHAD provide valuable insights into keratinocyte transcriptional reprogramming, these analyses cannot fully resolve dynamic changes during disease progression and spontaneous remission. Future longitudinal studies, ideally integrating multi-omics approaches, are likely to be essential to validate and extend these mechanistic insights.

In conclusion, our study underscores the role of keratinocytes in the pathogenesis of AD, particularly in immune responses and antigen presentation. In the SHAD group, a reduction in keratinocyte antigen presentation may facilitate skin repair, whereas the OVA mouse model, while providing valuable insights into immune responses, does not fully recapitulate this self-healing process, reflecting its limitations in modelling keratinocyte function and the chronic immune features of AD. Future research utilizing single-cell genomics is essential to identify more suitable animal models and further explore the role of keratinocytes, ultimately advancing targeted therapeutic strategies for AD.

## Supplementary Material

Supplemental Material

## Data Availability

The single-cell sequencing data for human skin tissue in this study are available in the NCBI Gene Expression Omnibus (GEO) under accession numbers GSE153760 and GSE162054. The single-cell transcriptomic data for mouse skin are deposited under accession code GSE194254. Data supporting the findings of this study are available from the corresponding author upon reasonable request.
