# Supplementary material for: Single-cell transcriptome revealed the aberrant keratinocytes activation in antigen presentation in atopic dermatitis
Source: Ann Med. 2026 Feb 10;58(1):2627742. doi: 10.1080/07853890.2026.2627742 (PMC12893161; doi:10.1080/07853890.2026.2627742)
Supplement: Supplemental Material [file IANN_A_2627742_SM3366.zip › suppl_data/Figure legends.docx]

**Supplementary Figure Legends**

**Figure S1 Single cell sequencing information, quality control results**. (A) Diagram of the sample preparation process for scRNA-seq of HC and AD skin samples. (B) Violin plots demonstrate the number of genes (nFeature_RNA) and unique molecular identifier (nCount_RNA) in each sample. (C) PCA of each sample. (D) UMAP plot color-coded by cell source of each sample. (E) The heatmap showed the top 5 differentially expressed genes (DEGs) of each cell cluster.

**Figure S2 Quality control and single-cell sequencing analysis of HC and AD samples.** (A) The pseudotime trajectory of keratinocyte population colored by two sample groups. (B) Cell percentages of four cell subtypes among two sample groups (left) and four cell subtypes among three states (right). (C) Venn diagram depicting the DEGs of four cell subtypes between two sample groups. (D) The volcano plot showing the DEGs of keratinocyte between two sample groups. (E) The bubble chart showing the GO enrichment results of DEGs in keratinocyte.

**Figure S3 Histological and physiological comparisons between CON and OVA groups.** (A) Mouse dorsal skin lesions of CON and OVA groups. (B) Representative H&E-stained images shows the differences between the CON and OVA-treated groups. Scale bar, 200 μm. (C-D) Representative images of spleen and thymus from the control and OVA-treated groups, illustrating the differences in organ morphology between the two groups. (E-F) The bar plot shows the comparison of skin elasticity scores and skin pH levels between the control and OVA-treated groups.

**Figure S4 Quality control and single-cell sequencing analysis of CON and OVA groups.** (A) Violin plots demonstrate nFeature_RNA, nCount_RNA, and percentage of mitochondria genes (percent.mt) in CON and OVA samples. (B) PCA of each sample. (C) UMAP plot color-coded by cell source of CON and OVA groups. (D) The volcano plot shows the log2 fold change (log2FC) on the y-axis and the percentage difference (Pct difference) between cell types on the x-axis.

**Figure S5 Differential gene functional analysis and cell communication between keratinocytes and immune cells in CON and OVA groups.** (A) The volcano plot showing the DEGs of keratinocyte between CON and OVA groups. (B) The top 8 GO terms from the GO analysis of DEGs. (C) The KEGG enrichment analysis of DEGs in keratinocytes between CON and OVA groups highlighted four significantly enriched key pathways. (D-E) Dot plot and chord charts of expression level of differentially up-regulated and down-regulated signal ligand-receptor pairs in keratinocytes and immune cell of CON and OVA groups.

**Figure S6 Differential gene functional analysis and cell communication between AD and SHAD groups.** (A) UMAP projection of single-cell profiles reveals 19 cell clusters. (B) Representative canonical cell type-specific marker expression across all cell clusters. (C) UMAP projection of single-cell profiles reveals 10 cell types. (D) GO enrichment analysis of 813 significantly upregulated genes between AD and SHAD groups. (E) Chord charts illustrate the expression levels of incoming signaling pathways (left) and outcoming signaling pathways (right) between five immune cell types and four keratinocyte subtypes in the SHAD group. (F) The violin plots show the expression levels of differentially regulated ligand-receptor pairs between five immune cell types and four keratinocyte subtypes in each group.

**Figure S7 Phenotypic analysis of spontaneously healed OVA (SHOVA) mice.**

(A) Schematic diagram illustrating the induction model for SHOVA group in mice. (B) Mouse dorsal skin lesions of SHOVA groups. (C) Representative H&E-stained images of the SHOVA groups. Scale bar, 200 μm. (D) The bar plot shows the comparison of epidermal thickness between the OVA and SHOVA groups. (E-F) The bar plot shows the comparison of spleen index and thymus index between the OVA and SHOVA groups. (G) The bar plot shows the comparison of skin moisture between the OVA and SHOVA groups. (H) Relative mRNA expression level of genes in each group.
