# Supplementary material for: Single-cell transcriptome revealed the aberrant keratinocytes activation in antigen presentation in atopic dermatitis
Source: Ann Med. 2026 Feb 10;58(1):2627742. doi: 10.1080/07853890.2026.2627742 (PMC12893161; doi:10.1080/07853890.2026.2627742)
Supplement: Supplemental Material [file IANN_A_2627742_SM3366.zip › suppl_data/Table S1.docx]

**Table S1. Primers list.**

| **Genes** | **Genbank** | **Forward primer sequence** | **Reverse primer sequence** | **Product Length (bp)** |
| --- | --- | --- | --- | --- |
| *Eif4a1*  *Rpl27a* | NM_001159375  NM_011975 | ATGTCTGCGAGTCAGGATTCT  TATCACCCAGGTTACTTTGGGA | AGCTATCCACAATCTCGTTCCA  ATGTCCACAGTTTATCCAGGTTG | 100  100 |
| *Gapdh*  Ndufa1  Cox6a1  Ndufa6 | [NM_008084](http://www.ncbi.nlm.nih.gov/entrez/query.fcgi?cmd=Search&db=Nucleotide&term=NM_008084)  NM_019443  NM_007748  NM_001033305 | AGGTCGGTGTGAACGGATTTG  ATGTGGTTCGAGATTCTCCCT  TCAACGTGTTCCTCAAGTCGC  TCGGTGAAGCCCATTTTCAGT | TGTAGACCATGTAGTTGAGGTCA  TGGTACTGAACACGAGCAACT  AGGGTATGGTTACCGTCTCCC  CTCGGACTTTATCCCGTCCTT | 123  131  115  148 |
