# Supplementary figures and images for: Single-cell transcriptome revealed the aberrant keratinocytes activation in antigen presentation in atopic dermatitis
Source: Ann Med. 2026 Feb 10;58(1):2627742. doi: 10.1080/07853890.2026.2627742 (PMC12893161; doi:10.1080/07853890.2026.2627742)

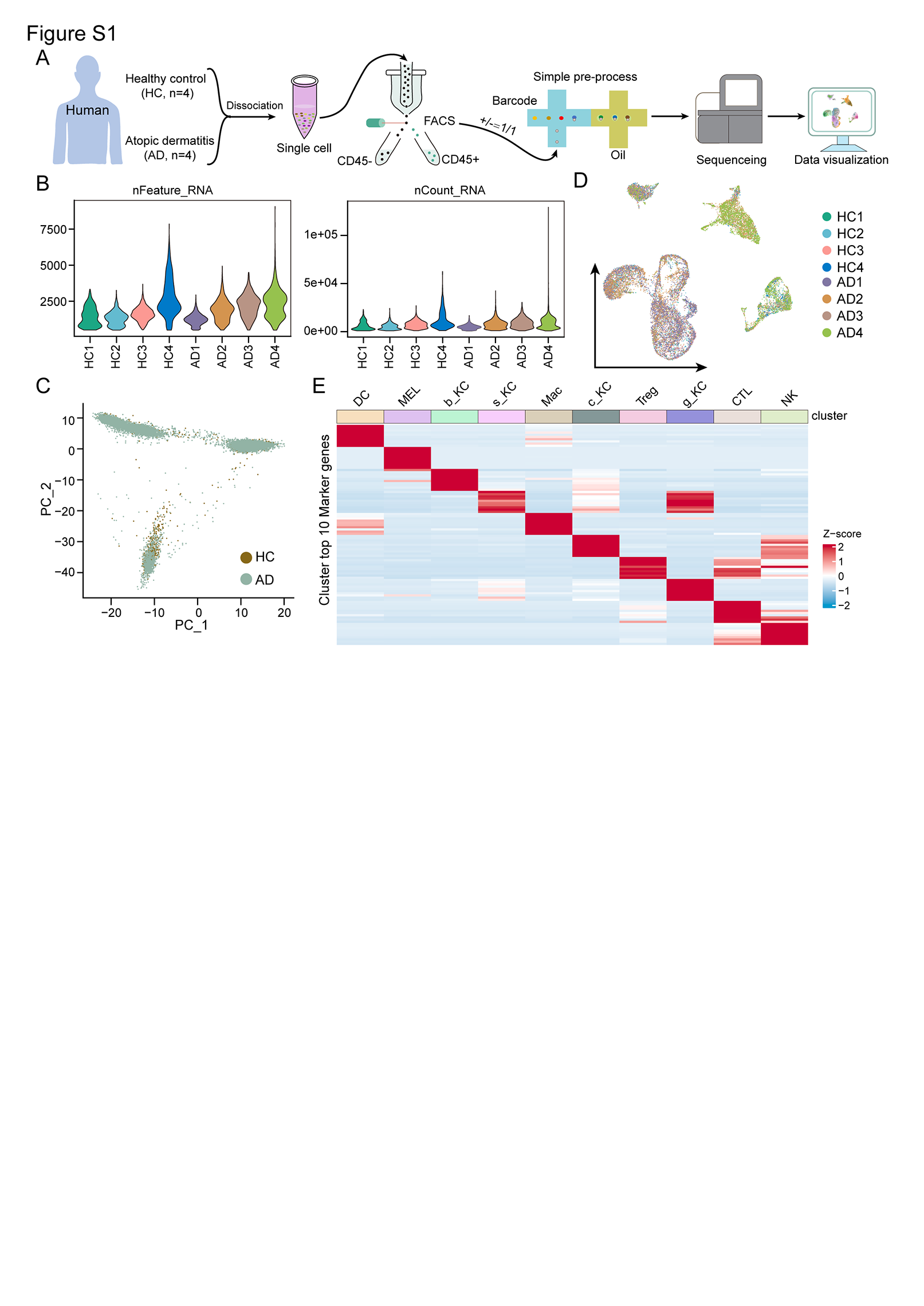

Supplement: Supplemental Material [file IANN_A_2627742_SM3366.zip › suppl_data/Fig S1.tif]

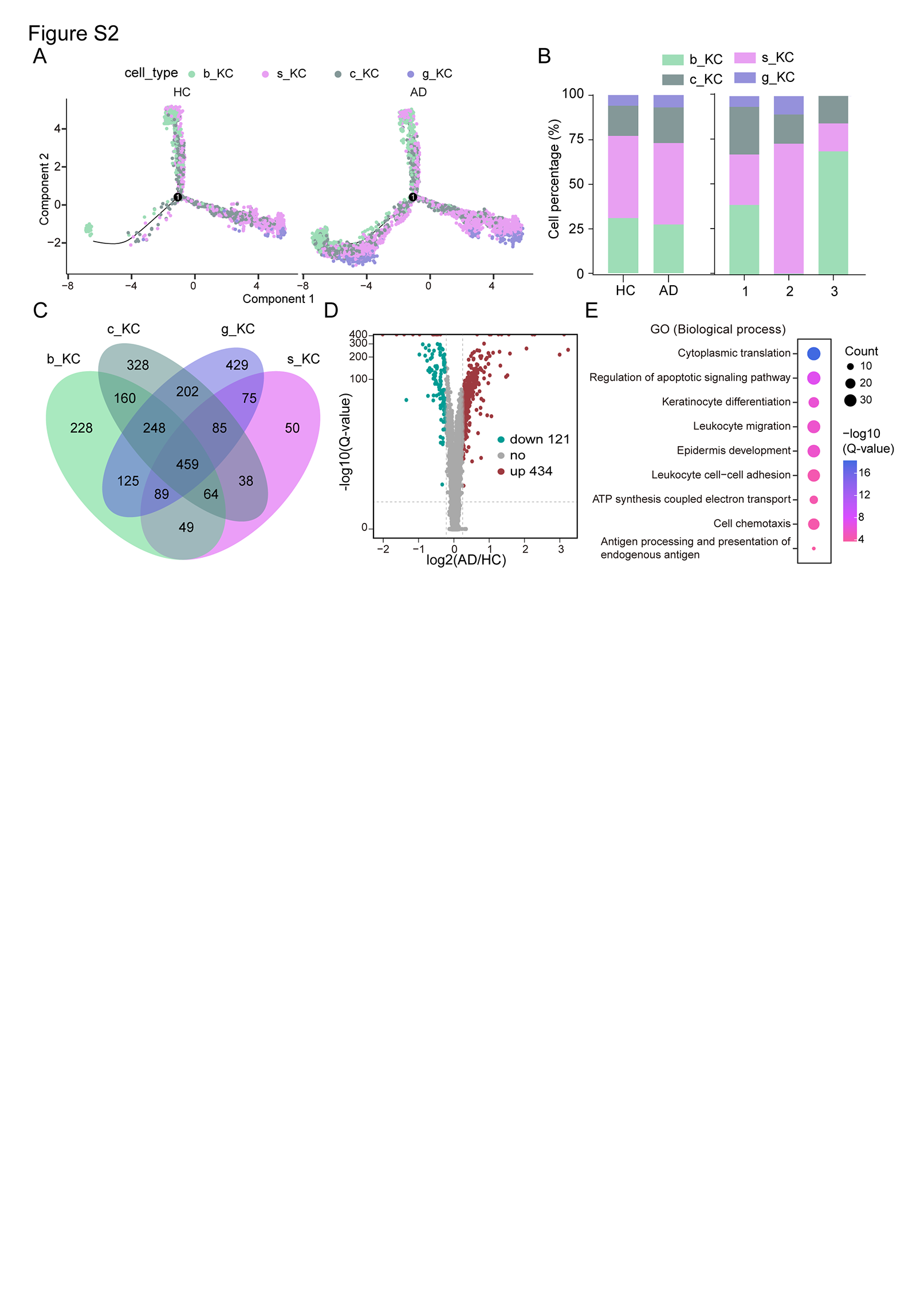

Supplement: Supplemental Material [file IANN_A_2627742_SM3366.zip › suppl_data/Fig S2.tif]

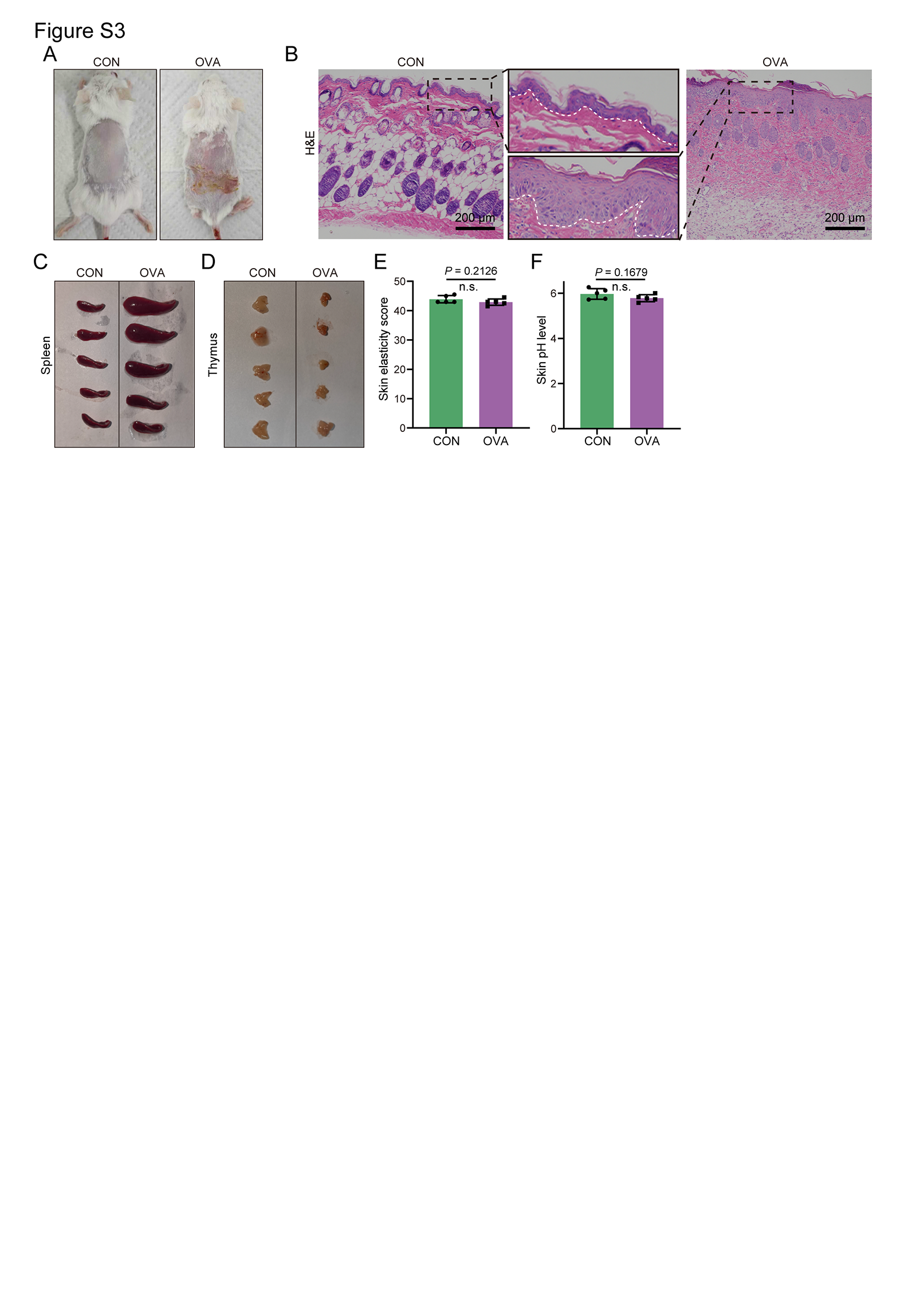

Supplement: Supplemental Material [file IANN_A_2627742_SM3366.zip › suppl_data/Fig S3.tif]

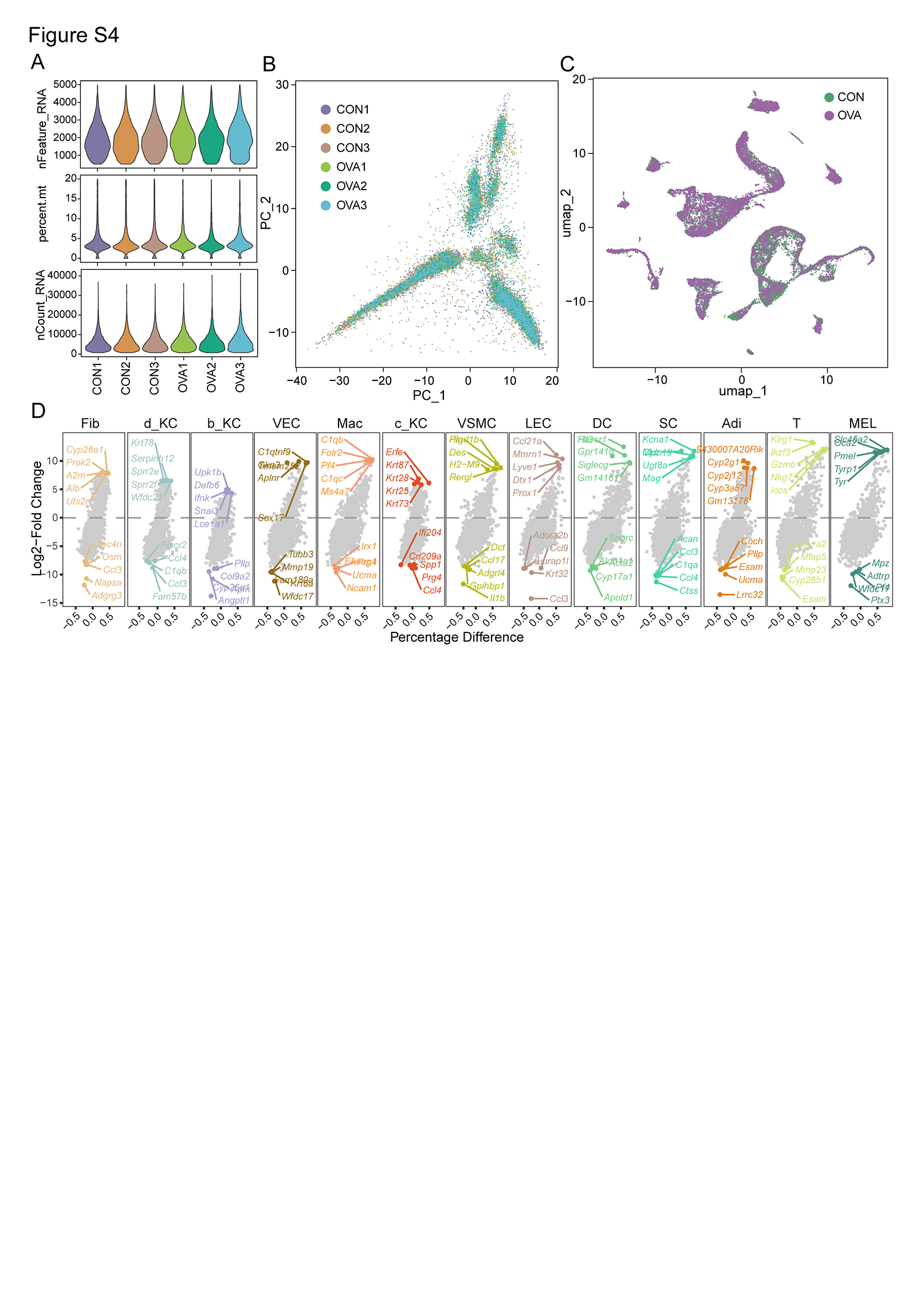

Supplement: Supplemental Material [file IANN_A_2627742_SM3366.zip › suppl_data/Fig S4.tif]

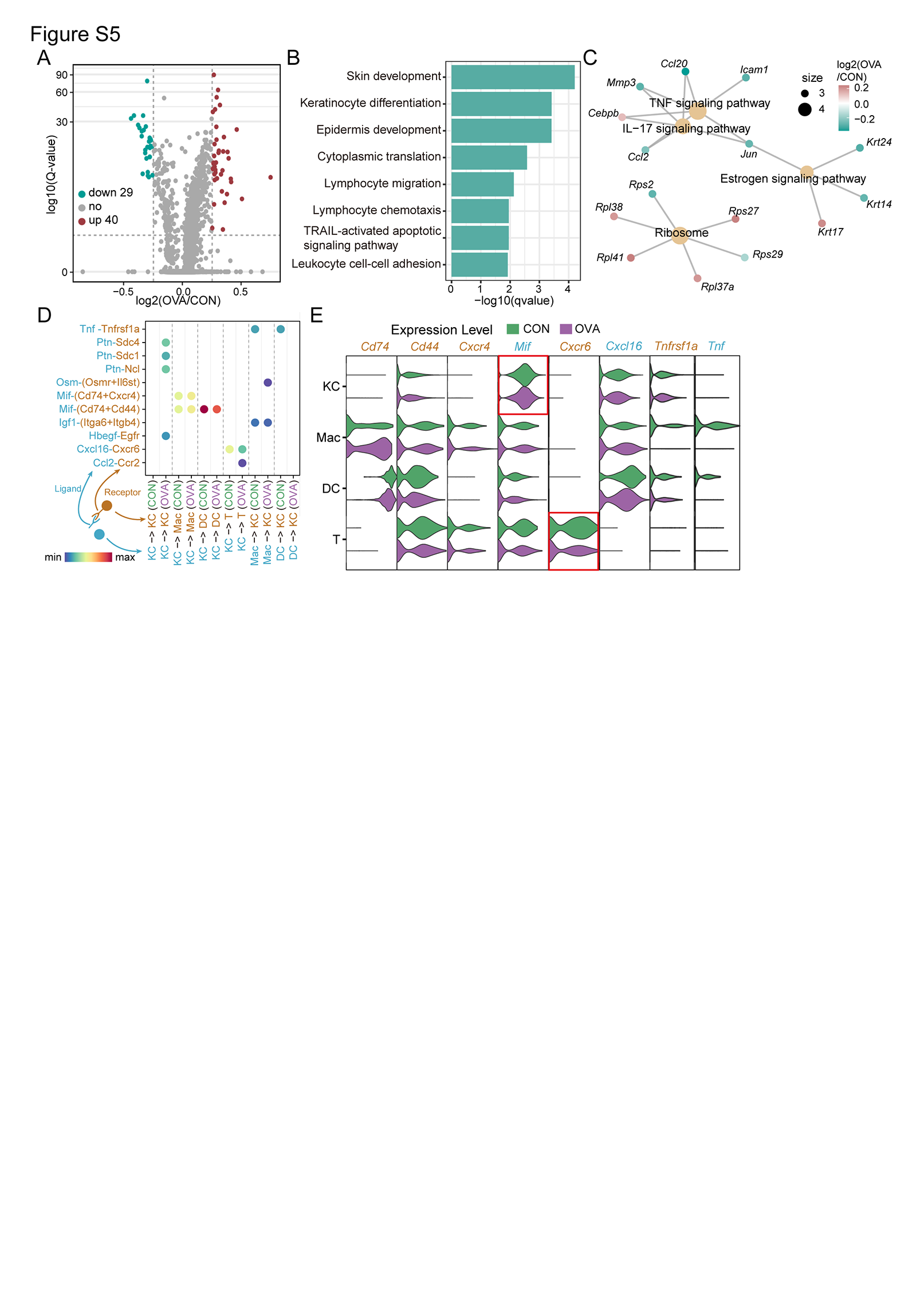

Supplement: Supplemental Material [file IANN_A_2627742_SM3366.zip › suppl_data/Fig S5.tif]

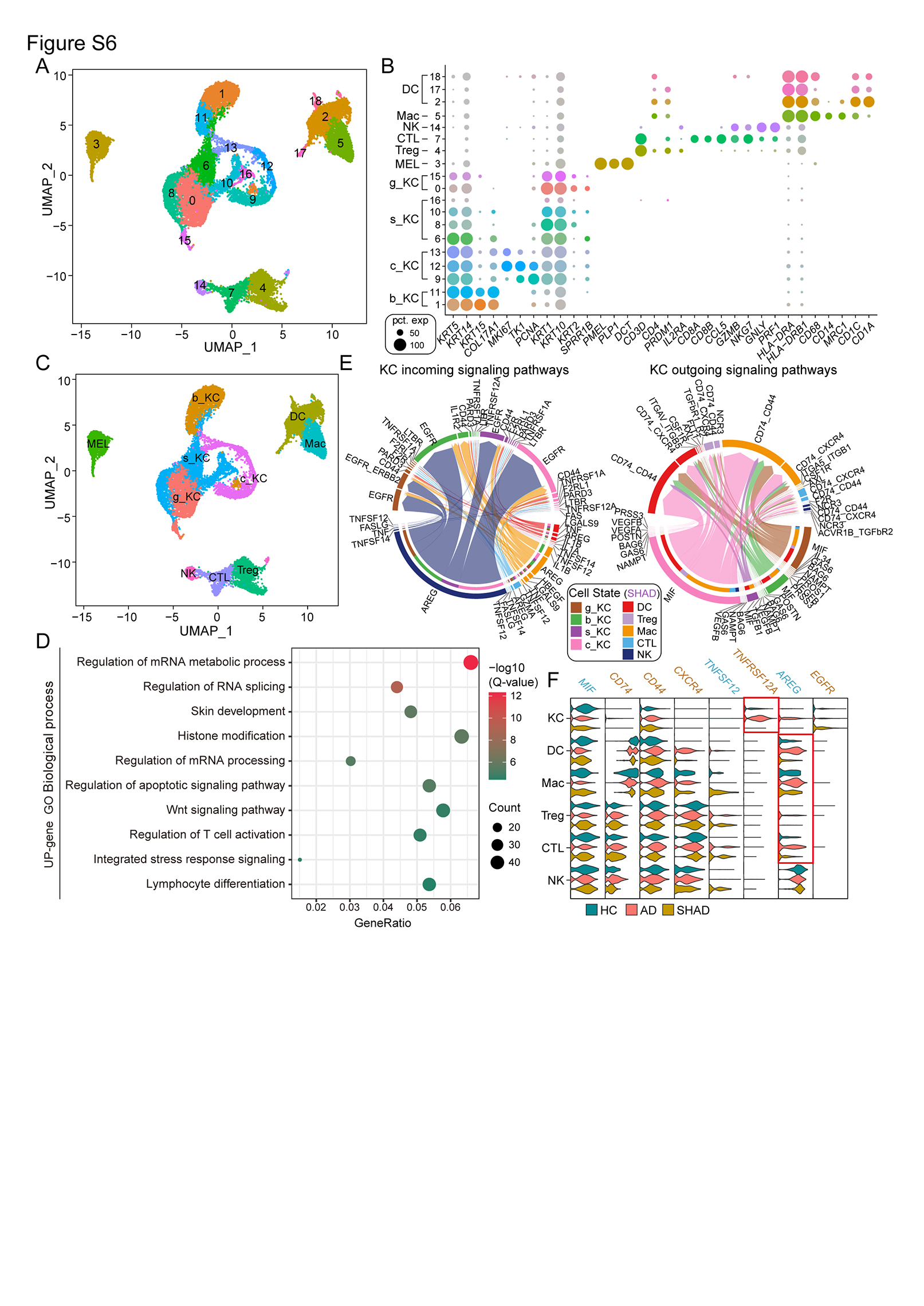

Supplement: Supplemental Material [file IANN_A_2627742_SM3366.zip › suppl_data/Fig S6.tif]

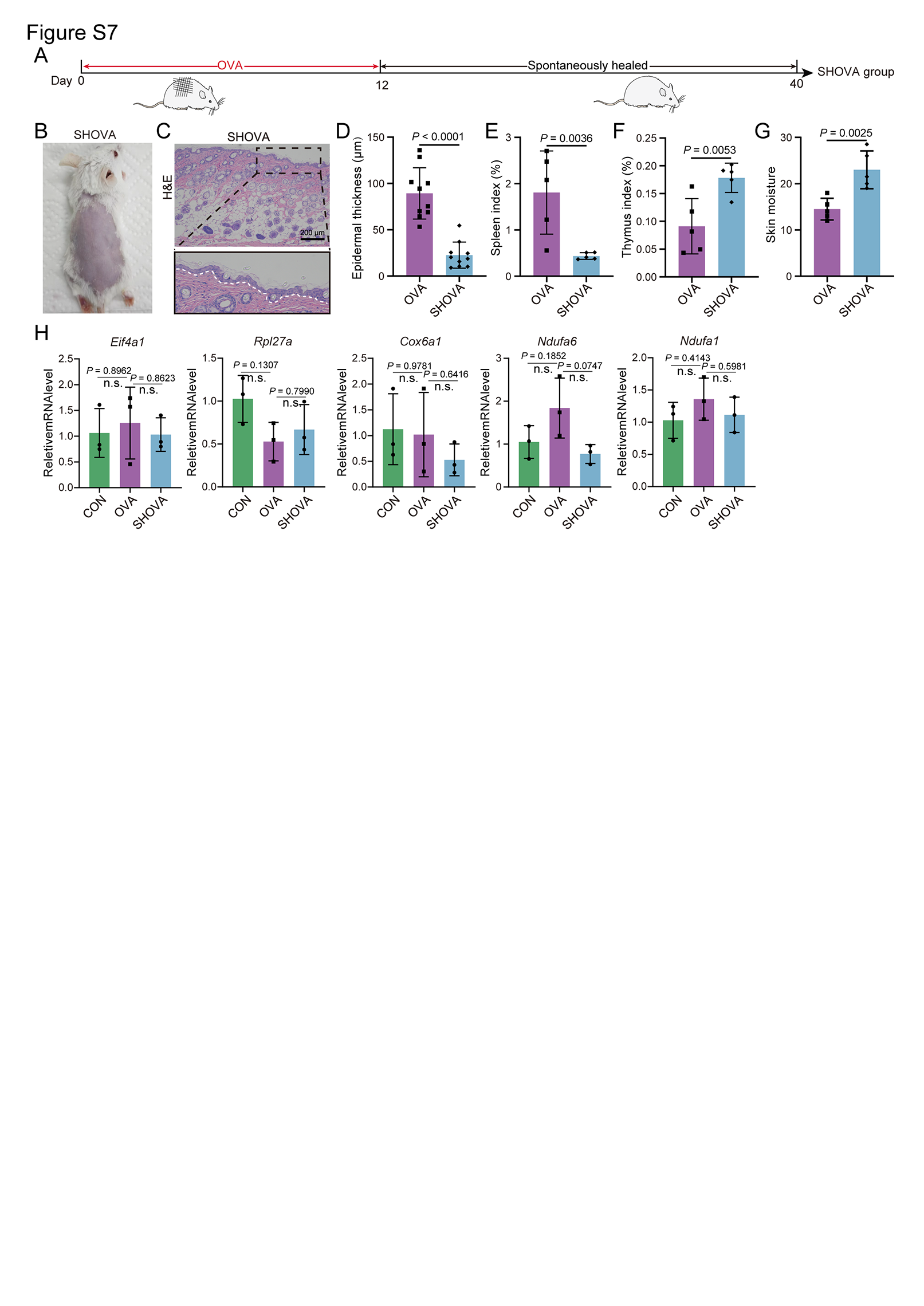

Supplement: Supplemental Material [file IANN_A_2627742_SM3366.zip › suppl_data/Fig S7.tif]
